# Retraction: Regulation of Cigarette Smoke (CS)-Induced Autophagy by Nrf2

**DOI:** 10.1371/journal.pone.0290862

**Published:** 2023-08-25

**Authors:** The PLOS ONE Editors

Following the publication of this article [1, 2], concerns were raised regarding Figures 3–5. Specifically,

In Figure 3B:

- In the p-mTOR panel:
  - Bands 1 and 5 appear similar when band 1 is horizontally flipped.
  - Band 1 appears to be flanked by vertical discontinuities when background levels are adjusted.
  - Lanes 1, 5, and 7 appear to have similar patterns within the background.
- In the Keap1 panel, lanes 2 and 5 appear to have similar patterns within the background.
- In the Actin panel, lanes 5 and 8 appear to have similar patterns within the background.
- In the S6K panel, lanes 2 and 8 appear to have similar patterns within the background.

In Figure 4B:

- In the p-mTOR panel, lanes 5 and 6 appear to have similar background patterns when lane 5 is horizontally flipped.

In Figure 5A:

- In the Actin panel:
  - Lanes 4 and 8 appear to have similar features within the background.
  - There appears to be a vertical discontinuity between lanes 7 and 8.

In Figure 5B:

- In the Actin panel, when levels are adjusted to visualize background, lanes 1 and 3 appear to have similar features within the background.

In Figure 5C:

- In the Actin panel:
  - Lanes 5 and 7 appear to have similarities in both the bands and background features.
  - Bands 6 and 8 appear to be similar.

The corresponding author stated that the underlying data for this article [1, 2] is no longer available. The corresponding author also stated that there is a cut between lanes 4 and 5 in the NQO1 panel and between lanes 7 and 8 in the Actin panel in Figure 5A. They stated that the cut between lanes 7 and 8 in the Actin panel in Figure 5A likely results from membrane damage.

In light of the above concerns with Figures 3B, 4B, and 5A-C which question the validity of these data, and in the absence of the underlying data required to resolve these concerns, the *PLOS ONE* Editors retract this article. YC & LZ did not agree with the retraction and stand by the article’s findings. ECB responded but neither agreed nor disagreed with the retraction. YX, ZL, and AM either did not respond directly or could not be reached.
